# Retinopathy of Prematurity: Historical Evolution of Clinical Management and Medico-Legal Evaluation in Japan

**DOI:** 10.3390/healthcare14101379

**Published:** 2026-05-18

**Authors:** Shigeo Iijima

**Affiliations:** Department of Regional Neonatal-Perinatal Medicine, Hamamatsu University School of Medicine, Hamamatsu 431-3192, Japan; siijima@hama-med.ac.jp; Tel.: +81-53-435-2312

**Keywords:** retinopathy of prematurity, neonatal intensive care, patient safety, healthcare quality, risk management, standard of care, informed consent, medical litigation, oxygen therapy, anti-VEGF therapy

## Abstract

**Highlights:**

**What are the main findings?**

- Judicial evaluations of retinopathy of prematurity-related malpractice in Japan shifted markedly after 1975, reflecting the nationwide dissemination of screening and photocoagulation practices.
- Courts defined the “standard of care” not solely by published evidence but also by the state of professional knowledge, institutional capabilities, and available treatment options at the time.

**What are the implications of the main findings?**

- Historical retinopathy of prematurity-related litigation demonstrates how rapidly evolving neonatal technologies can create patient safety risks when system-level standards are not yet fully established.
- In the era of anti-vascular endothelial growth factor therapy, transparent treatment selection, structured referral systems, and robust informed consent processes are critical for reducing medicolegal risk and improving healthcare quality.

**Abstract:**

The management of retinopathy of prematurity (ROP) has evolved alongside advances in neonatal medicine, shaped by both scientific progress and medico-legal influences. ROP-related medical malpractice lawsuits provide a unique perspective on how standards of care are defined and evaluated. This review comprises two complementary components: a narrative medico-historical review of ROP management and a structured descriptive comparison of judicial cases identified in publicly accessible legal databases in Japan. The aim was to clarify how evolving clinical practice, dissemination of knowledge, and institutional capacity have influenced judicial interpretations of the standard of care. The findings indicate that standards of care in ROP management have been determined not solely by established evidence but by a broader assessment of contemporaneous professional knowledge and clinical practice. A marked shift in judicial outcomes after the mid-1970s corresponded to the widespread adoption of systematic screening and photocoagulation therapy. These results suggest that medico-legal evaluation reflects system-level maturity in neonatal care. In the current era of anti-vascular endothelial growth factor therapy, litigation is likely to focus less on specific interventions and more on the appropriateness of clinical decision-making, consideration of alternatives, and adequacy of informed consent procedures. This review provides a medico-historical framework for improving patient safety, risk management, and quality of care in neonatal practice.

## 1. Introduction

Advances in neonatal care have markedly improved the survival rate of preterm infants worldwide, leading to an increasing burden of prematurity-associated morbidities [1]. Retinopathy of prematurity (ROP) is a vasoproliferative disease that affects the incompletely vascularized retinas of preterm infants and remains a major cause of childhood blindness globally [2]. The risk and severity of ROP are strongly associated with lower birth weight and gestational age [3].

ROP emerged as a major clinical problem in the mid-20th century, following the widespread adoption of oxygen therapy and incubator-based neonatal care [4]. Subsequent studies established a strong association between supplemental oxygen and ROP development, leading to major changes in oxygen management practices [5,6]. In the United States, this period was also characterized by numerous medical malpractice lawsuits related to ROP, in which excessive oxygen administration and inadequate monitoring were frequently contested, contributing to the development of the medico-legal framework of neonatal care [7].

In Japan, ROP became a prominent clinical and social issue in the 1960s, later than in Western countries. The number of ROP-related medical malpractice lawsuits increased rapidly in the early 1970s, peaking in 1974 [8]. These cases, filed nationwide, represent one of the largest waves of medical litigation in the history of the Japanese healthcare system and involve retrospective evaluations of neonatal care under rapidly evolving medical knowledge and limited clinical infrastructure.

From a healthcare system perspective, ROP represents a paradigmatic condition in which rapidly evolving technologies and clinical practices may outpace the dissemination of standardized care, resulting in preventable patient harm and medicolegal risk. Examining how standards of care were historically defined and evaluated in ROP-related litigation may provide important insights into contemporary patient safety, risk management, and quality improvement in neonatal care. To address this, the present study adopts a dual methodological approach, integrating a narrative medico-historical review of clinical developments with a structured descriptive comparison of judicial cases.

This narrative review integrates a historical analysis of ROP management with a structured descriptive analysis of related judicial cases. The aim was not to provide a formal epidemiological evaluation but to clarify how standards of care have evolved through the interaction between medical practice and legal assessment. Specifically, this study aimed to (1) summarize the historical development of ROP diagnosis and treatment, (2) examine the evolution of ROP-related litigation in the United States and Japan, and (3) analyze how courts have evaluated contemporaneous standards of care across different periods. Through an integrated medico-historical approach, this study sought to provide a framework for understanding current challenges in ROP management, particularly regarding treatment decision-making, institutional responsibility, and informed consent.

## 2. Methods

This study is structured into two methodologically distinct, yet conceptually integrated components: (1) a narrative medico-historical literature review of the development of ROP management; and (2) a structured descriptive comparison of judicial cases related to ROP. These components, which follow distinct criteria, data sources, and analytical procedures, are presented as independent methodological components with distinct structures, yet integrated to examine how the standard of care has evolved and been interpreted amid changing clinical knowledge.

### 2.1. Literature Review

A literature review was conducted to identify key studies relevant to the historical development of ROP management. PubMed and Google Scholar were searched through January 2026 using combinations of keywords including “retinopathy of prematurity,” “oxygen therapy,” “screening,” “photocoagulation,” “cryotherapy,” “anti-VEGF,” “medical malpractice,” and “standard of care.” Additional sources were identified by screening reference lists of key publications.

Considering the aim of this study, the literature review was not intended to be exhaustive but rather to support a structured narrative synthesis of major clinical, historical, and conceptual developments relevant to ROP and its medico-legal evaluation. Particular emphasis was placed on studies that represent key transitions in the understanding and management of ROP, especially during the postwar period when important shifts in clinical practice and knowledge dissemination occurred.

For methodological transparency, literature selection was guided by the following conceptual criteria: (1) relevance to key transitions in the clinical understanding and management of ROP; (2) contribution to the development, dissemination, or standardization of clinical practices; and (3) relevance to the evaluation of the standard of care in medico-legal contexts.

This approach ensures a structured and reproducible narrative synthesis, while maintaining flexibility appropriate for a medico-historical analysis.

### 2.2. Judicial Cases Analysis

This component is methodologically distinct from the literature review and was conducted using an independent procedure for data identification and analysis.

Japanese ROP-related malpractice cases were identified using publicly accessible legal databases, including D1-law.com (Daiichihoki, Tokyo, Japan) [9] and Westlaw Japan (Thomson Reuters, Tokyo, Japan) [10]. Publicly available judicial decisions were screened and included when the case clearly involved ROP/retrolental fibroplasia (RLF) and neonatal management issues relevant to ROP (e.g., oxygen administration, ophthalmologic screening, treatment, referral/transfer, or duty of explanation). From each decision, the following variables were extracted when explicitly described: infant birth year, alleged clinical issues, and judicial outcomes at the first instance and final adjudication. In group litigation, each infant was analyzed as an individual case when sufficient information was available from judicial records. Only publicly available judicial decisions were analyzed; no individual medical records or identifiable personal data were accessed.

Judicial decisions were analyzed because they provide a structured external evaluation of clinical practice under real-world conditions, particularly in situations where medical standards are evolving. This approach provides insight into how the standard of care is interpreted and applied in practice beyond purely scientific evidence.

### 2.3. Analytical Approach

The judicial case component is a structured descriptive comparison rather than a formal epidemiological or inferential statistical study.

Cases were stratified by birth year into two periods (≤1974 vs. ≥1975), reflecting the period before and after the nationwide dissemination of modern ROP management in Japan. Descriptive summaries were used to characterize case characteristics and judicial outcomes. Where appropriate, simple comparative assessments, including basic statistical tests (e.g., Student’s *t*-test or χ^2^ test), applied in an exploratory manner, were conducted using evaluable cases to elucidate differences between groups. Analyses were conducted using IBM SPSS Statistics (version 31; IBM Corp., Armonk, NY, USA). A post hoc power assessment was also performed to support the interpretation of observed differences.

These analyses provide contextual and comparative insights that support interpretative discussion, rather than establishing causal relationships.

## 3. ROP Pathophysiology

**Figure 1:**
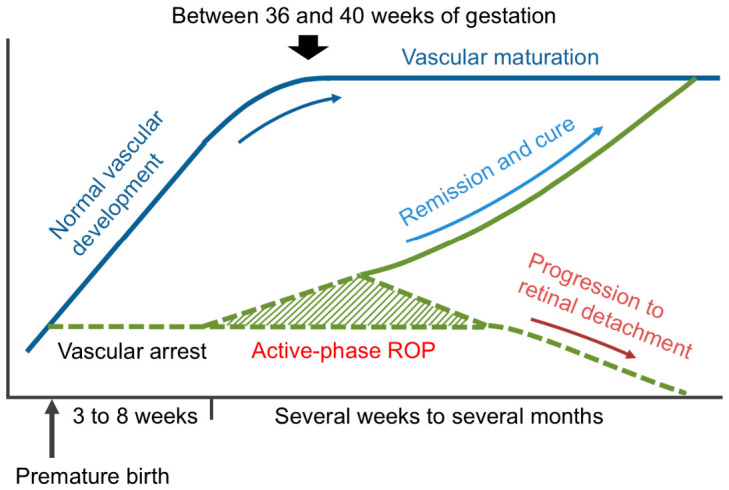
Development and progression of ROP. Abbreviation: ROP, retinopathy of prematurity.

In cases of normal fetal development, retinal vascularization begins at approximately 14 weeks of gestation at the optic disc and extends toward the peripheral retina. This process is largely completed by 36–40 weeks of gestation; accordingly, retinal vascular development is almost complete at birth in full-term infants [11] (Figure 1). In contrast, preterm infants are born with incompletely vascularized retinas, leaving avascular areas in the periphery.

When the retinal vessels have already extended relatively far toward the periphery at birth, physiological vascular growth may continue postnatally. However, in infants born at an earlier stage of vascular development, the abrupt transition from the intrauterine to the extrauterine environment, particularly exposure to a relatively hyperoxic postnatal milieu with suppressed vascular endothelial growth factor (VEGF) expression, can interrupt normal retinal vascularization [12]. This stage corresponds to phase I of ROP [13,14].

Subsequently, the avascular retina becomes relatively hypoxic, upregulating angiogenic factors, including VEGF, thereby leading to pathological neovascularization at the junction between the vascularized and avascular retina. This process corresponds to phase II of ROP [13,14].

Neovascular activity is often transient and may undergo spontaneous regression. However, in some infants, abnormal vessels fail to reestablish adequate retinal perfusion and evolve into extraretinal fibrovascular proliferation that extends into the vitreous. Contraction of these membranes exerts traction on the retina, potentially leading to tractional retinal detachment and severe visual impairment or blindness [15,16] (Figure 1).

Infants with lower gestational age and birth weight have more extensive avascular retinal areas and higher levels of angiogenic drive, placing them at an increased risk of developing severe forms of ROP.

## 4. Postwar Global Epidemic of ROP and Oxygen Management

**Figure 2:**
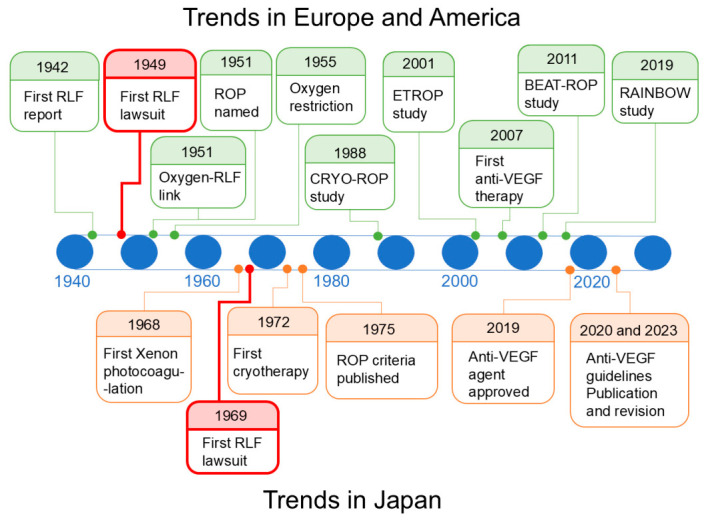
Simplified timeline of key medical and medico-legal milestones in the evolution of retinopathy of prematurity. The upper panel illustrates major global developments in ROP pathophysiology, monitoring, and treatment. The lower panel summarizes key milestones in Japan, including changes in clinical practice and litigation trends. Abbreviations: RLF, retrolental fibroplasia; ROP, retinopathy of prematurity; anti-VEGF, anti-vascular endothelial growth factor; Laser, laser photocoagulation.

Following World War II, advances in neonatal care, particularly the widespread use of supplemental oxygen and the introduction of closed incubator systems, substantially improved the survival of preterm and low-birth-weight infants. Although very low-birth-weight infants, particularly those weighing < 1000 g, rarely survived despite intensive care, more mature premature infants often survived. However, the improvement in survival was accompanied by a rapid increase in the incidence of severe visual impairment and blindness due to ROP, then termed RLF [17,18]. Accordingly, ROP/RLF emerged as a major clinical problem in Europe and North America during the late 1940s and early 1950s [19,20]. The historical evolution of ROP is shown in Figure 2.

A defining feature of neonatal practice during this period was the lack of reliable methods to objectively monitor oxygen concentration and exposure. Consequently, high concentrations of supplemental oxygen were administered for prolonged periods with minimal restrictions [21]. Based on the prevailing belief that oxygen is universally beneficial, inspired oxygen concentrations exceeding 70% and, in some cases, approaching 85%, were used continuously for weeks.

Subsequent clinical and experimental studies have demonstrated a strong association between excessive oxygen exposure and the development of ROP [5,6,22]. In response, oxygen restriction policies were introduced in the 1950s, with recommendations to limit inspired oxygen concentrations to ≤40% [17]. These measures resulted in a marked reduction in the incidence of severe ROP and ROP-related blindness.

However, strict oxygen restriction was later associated with increased mortality and morbidity from neonatal respiratory disorders, particularly respiratory distress syndrome [23]. Moreover, it was suggested that the reduction in ROP incidence was accompanied by an inverse increase in both the frequency and severity of cerebral palsy [24]. This prompted a re-evaluation of oxygen management strategies, leading to more liberal oxygen use under specific clinical conditions [25,26].

With the continued evolution of neonatal intensive care in the late 1960s and 1970s [27], including the introduction of oxygen monitoring technologies and improved survival of extremely preterm infants, a renewed increase in ROP incidence was observed, often referred to as the “second epidemic” [28].

These historical transitions illustrate a fundamental challenge in neonatal medicine: balancing the benefits of life-saving interventions against the risk of iatrogenic complications. Importantly, this dynamic clinical uncertainty also shaped medico-legal evaluations, as standards of care were defined not by fixed criteria but by the evolving interplay between available scientific evidence, clinical practice, and technological capacity at a given time.

## 5. Trends in ROP Management

The management of ROP evolved from observational approaches to structured screening and active intervention, reflecting advances in clinical knowledge and therapeutic capabilities.

Early efforts focused primarily on identifying at-risk infants and monitoring disease progression. During the 1960s, recommendations emerged emphasizing the importance of ophthalmologic examination in premature infants, particularly those exposed to supplemental oxygen [29]. These early initiatives laid the foundation for systematic screening programs. Over time, screening criteria became standardized based on birth weight, gestational age, and clinical condition, and regular funduscopic examination became an essential component of neonatal care [30,31].

In parallel, various pharmacological therapies were explored for ROP management, including vitamin E, adrenocorticotropic hormone, and corticosteroids [32,33,34]. Although some studies suggested potential benefits [35,36,37], the results were inconsistent and inconclusive [38,39,40,41]. The high rate of spontaneous regression in ROP further complicated the evaluation of therapeutic efficacy [42], and ethical constraints limited the feasibility of controlled clinical trials. Consequently, pharmacological treatments were not established as effective interventions and were gradually abandoned.

A major turning point in ROP management occurred with the introduction of interventional therapies [43]. Retinal photocoagulation, first reported in 1968 [44], and cryotherapy, introduced shortly thereafter [45], were the first treatments to demonstrate clinical efficacy in preventing disease progression. Large-scale studies in the 1980s, including the CRYO-ROP trial, confirmed the effectiveness of cryotherapy [46], leading to its temporary adoption as a standard treatment [47]. Subsequently, laser photocoagulation replaced cryotherapy due to the superior safety profile and clinical outcomes, becoming the preferred treatment modality [48,49].

These developments marked a transition from passive observation to active treatment and defined the point at which effective therapeutic intervention became available. This transition is of particular importance in medico-legal contexts, as the availability and dissemination of effective treatments directly influenced how the standard of care was interpreted in clinical practice and evaluated in judicial settings.

## 6. Development of ROP-Related Medical Litigation in the United States

This section examines how evolving clinical uncertainty in neonatal care was reflected in legal reasoning and judicial decision-making in the United States.

During the 1940s and 1950s, as the number of children blinded by ROP, then termed RLF, increased markedly, clinicians struggled to determine the appropriate dose and duration of supplemental oxygen therapy. In this climate of clinical uncertainty, lawsuits alleging medical malpractice began to emerge throughout the United States [50].

In the 1950s, the American Academy of Pediatrics oxygen administration guidelines, which recommended limiting inspired oxygen concentration to ≤40%, were widely regarded as the de facto standard of care in neonatal practice. These guidelines subsequently played a pivotal role in ROP-related litigation. Following the first reported malpractice in 1949 [17], the central issue, in many cases, was whether the concentration or duration of oxygen administration was excessive. Notably, the use of inspired oxygen at concentrations exceeding 40% was frequently associated with negligence.

Although widespread adoption of oxygen restriction (FiO_2_ ≤ 0.40) led to a marked decline in newly diagnosed ROP, it later became evident that the disease could still occur, albeit infrequently, even in the absence of substantial oxygen exposure [51]. Consequently, disputes regarding the causal relationship between brief postnatal oxygen administration and subsequent ROP development continued [7]. Moreover, concerns that strict oxygen restriction might have increased mortality from respiratory distress syndrome [23] further complicated the judicial determination of causation.

In ROP-related malpractice actions, juries commonly focused on three principal issues: (1) whether the care provided conformed to the standard of care prevailing at the relevant time; (2) whether a causal link between oxygen administration and the development of ROP could be established; and (3) whether adequate disclosure and informed consent had been obtained from the infant’s parents. For example, a New York jury in 1976 concluded that the oxygen practices used in 1954 were consistent with generally accepted standards, resulting in a verdict in favor of the defendants. In contrast, in a case involving a premature infant born in 1953 (birth weight, 1362 g), high-concentration oxygen was found to have been administered for partially investigational purposes without sufficient medical necessity and without adequate explanation to the parents, and both negligence and lack of informed consent were determined [52]. During the same period, reports of substantial financial settlements were also published [53], highlighting the variability in outcomes. Although the profound emotional impact of childhood blindness might have influenced jury decisions, legal commentators have emphasized the importance of careful, evidence-based adjudication grounded in medical science [54]. These cases illustrate that legal judgments were shaped not only by clinical outcomes but also by the evolving understanding of acceptable medical practice at the time.

## 7. Postwar Trends and Judicial Evaluation of ROP in Japan

This section analyzes how Japanese courts evaluated clinical practices for the management of ROP amid evolving medical knowledge and limited clinical infrastructure.

### 7.1. ROP in Japan from the Postwar Period to the 1980s

Before World War II, neonatology was not established as a distinct medical discipline in Japan. Prolonged postwar disruptions further delayed the introduction of modern knowledge and technology for the care of premature infants, resulting in a substantial lag compared with Western countries. In the immediate postwar period, makeshift devices were used in place of incubators, and systematic neonatal intensive care began to develop only in the late 1940s [55]. The first closed-type incubator was introduced in Japan in 1954, and organized care for low-birth-weight infants gradually emerged around 1960.

The first Japanese report of ROP, then termed RLF, was published in 1949, 7 years after its initial description in Western countries [18,56]. However, in contrast to those from Europe and North America, early reports from Japan described relatively few severe cases. This was attributed to the limited use of high-concentration oxygen and underdeveloped neonatal care systems at the time.

From the late 1950s, the use of closed incubators and supplemental oxygen gradually increased nationwide. Although international experience had already emphasized cautious oxygen administration, the limited availability of arterial oxygen monitoring led to variability in clinical practice. As a result, a resurgence of ROP cases was observed from the late 1960s to the early 1970s. Efforts to detect ROP through funduscopic examination began in the mid-1960s [57], initially as exploratory and research-oriented activities conducted by a limited number of clinicians. Longitudinal observations revealed that a substantial proportion of premature infants developed ROP, with most cases regressing spontaneously but a minority progressing to visual impairment or blindness [58]. These findings highlighted the need for systematic screening and early intervention [55].

During the 1970s, screening practices gradually expanded, although their dissemination was limited by technical challenges and the need for collaboration between ophthalmologists and neonatologists.

With the introduction of photocoagulation therapy, fundus examination became essential not only for monitoring oxygen-related effects but also for determining treatment indications and optimal timing. In 1977, the Japan Pediatric Society issued recommendations for systematic ROP screening based on birth weight, gestational age, and oxygen exposure, representing a major step toward nationwide standardization [59]. Simultaneously, pharmacological therapies such as adrenocorticotropic hormone and corticosteroids were attempted, but without consistent clinical success. Interventional treatments, particularly photocoagulation, were rapidly adopted in Japan following the early pioneering work [44]. The introduction of pulse oximetry in the late 1970s further improved oxygen management and contributed to a decline in ROP cases [8].

These developments collectively established the clinical framework for ROP management in Japan by the mid-1970s and provided a foundation for subsequent medico-legal evaluation of neonatal care practices.

### 7.2. Development of ROP-Related Medical Malpractice Litigation in Japan

The earliest ROP-related lawsuit in Japan was filed with the Osaka District Court in 1969 and involved a premature infant born in 1967 who developed bilateral blindness following supplemental oxygen therapy. The central legal issues were whether oxygen therapy had been prolonged without sufficient medical indication after clinical stabilization and whether the absence of ophthalmologic examinations prevented timely diagnosis and intervention. At that time, although excessive and prolonged oxygen exposure was recognized as a potential risk factor for ROP, the safety of lower oxygen concentrations had not been firmly established, and methods for fundus examination in premature infants were not yet standardized [57]. In this context, the court determined that decisions regarding oxygen management fell within the physicians’ clinical discretion and that routine ophthalmologic screening could not yet be considered an established obligation. Consequently, negligence was not recognized. Similar provider-favorable judgments followed, reflecting judicial reliance on the contemporaneous level of medical knowledge.

A major shift occurred with a landmark patient-favorable decision by the Gifu District Court in 1974. In this case, the court conducted a comprehensive evaluation of neonatal management, including oxygen administration, timing of ophthalmologic examinations, assessment of disease severity, therapeutic decision-making, and duty of explanation. Although negligence in oxygen administration was not established, the court identified breaches related to delayed examination, misjudgment of severity, inappropriate timing of treatment, and inadequate informed consent. This decision marked a turning point in judicial attitudes and had a substantial impact on both medical practice and subsequent litigation.

**Figure 3:**
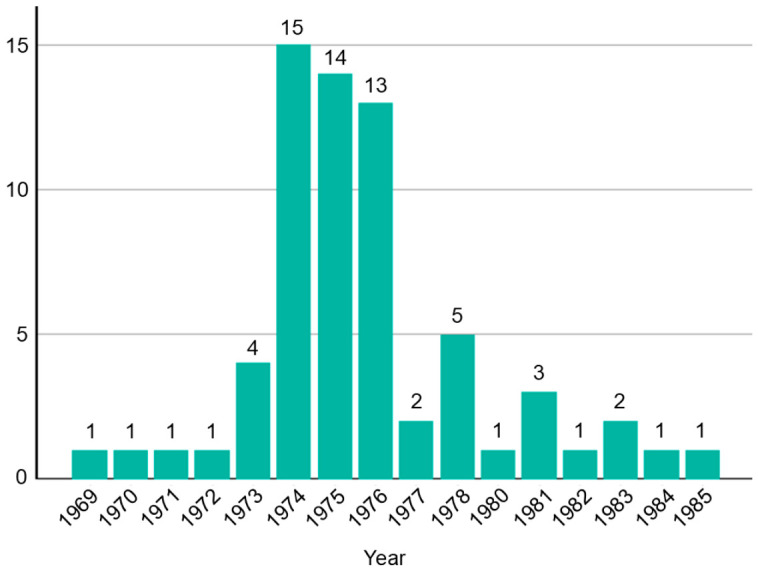
Annual number of retinopathy of prematurity-related medical malpractice lawsuits filed in Japanese district courts. White bars indicate the number of lawsuits filed each year. Numbers above each bar represent the total number of lawsuits filed in the corresponding year. Data were derived from publicly available judicial decisions [9,10].

Following this case, ROP-related litigation increased nationwide (Figure 3). A total of 66 cases involving 146 infants proceeded to first-instance judgments, including several group actions. The principal issue across these cases included the foreseeability of ROP, adequacy of systemic management, oxygen administration, implementation of regular fundus examinations, appropriateness of treatment, referral practices, and duties of explanation. In evaluating these issues, courts consistently applied the concept of “standard of care at the time of treatment,” emphasizing the importance of contemporaneous medical knowledge and practice.

**Figure 4:**
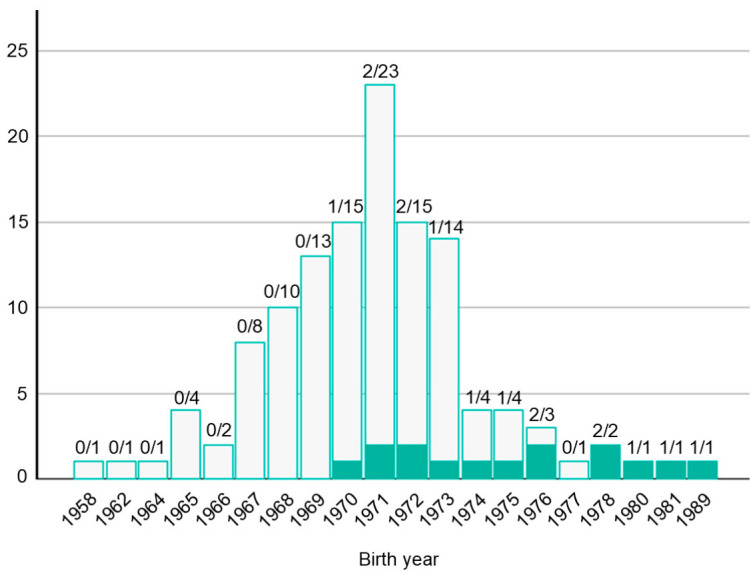
Distribution of Japanese retinopathy of prematurity-related malpractice cases and proportion of plaintiff-favorable judgments by birth year cohort. Bars show the total number of infants/cases with identifiable outcomes in publicly available judicial decisions for each birth year; the numerator/denominator above each bar indicates plaintiff-favorable outcomes/total outcomes (evaluable cases). In group litigation, each infant was analyzed as an individual case when sufficient information was available; therefore, counts in this figure do not necessarily correspond to the number of filed lawsuits, and the number of infants included from group actions may differ from the total group size due to incomplete descriptions in some decisions.

To further characterize temporal changes in judicial evaluation, a comparative analysis was conducted by birth cohort (≤1974 vs. ≥1975). Among cases in which individual-level data were available (Figure 4), provider-favorable judgments were recorded for 93.7% of infants born before 1974, compared with 38.5% in those born in 1975 or later (Table 1). In addition, the frequency with which courts recognized breaches related to key clinical issues—such as delayed ophthalmologic examination, inadequate treatment, and insufficient explanation—was higher in the latter cohort. These findings indicate that judicial interpretations of the standard of care evolved in parallel with the dissemination of clinical knowledge and the establishment of effective screening and treatment strategies. In particular, the widespread adoption of photocoagulation and the institutionalization of systematic screening in the mid-1970s [60] appear to have redefined the threshold for judging clinical practice negligent.

Furthermore, Supreme Court decisions introduced an important refinement to the standard of care, emphasizing that it should not be applied uniformly across all medical institutions. Instead, the expected standard depends on the level of knowledge and capability reasonably attributable to institutions of similar function and context. This interpretation placed greater responsibility on core perinatal centers and increased the likelihood of patient-favorable judgments or settlements in later cases.

Taken together, these analyses demonstrate that ROP-related litigation in Japan was not merely a reflection of clinical outcomes but rather a dynamic process shaped by the interplay among evolving medical knowledge, clinical practice, and judicial interpretation.

## 8. Contemporary Challenges in ROP Management and Potential Legal Implications

Currently, with the increasing chances of survival, the rates of ROP and severe ROP remain high among infants with extremely low birth weight and extremely low gestational age [61]. In these infants, prolonged supplemental oxygen therapy and the higher frequency of systemic comorbidities increase the risk of ROP.

The management of ROP has undergone a major transition with the introduction of anti-VEGF therapy in the 2000s. Originally developed for oncologic use [62], anti-VEGF agents were subsequently applied to intraocular neovascular diseases and have increasingly been used in ROP [63].

In Japan, these agents were initially used off-label, although after large international trials, including the ranibizumab versus laser therapy for the treatment of very low-birth-weight infants with ROP (RAINBOW) study, ranibizumab and aflibercept were approved for ROP in 2019 and 2022, respectively. Compared with laser photocoagulation, which irreversibly ablates the peripheral retina and arrests physiological vascular growth, anti-VEGF therapy preserves the retinal tissue and may allow continued vascular development after treatment [64]. It has also been reported to be effective in certain advanced cases [65] and may induce more rapid regression of neovascular activity [66]. However, the optimal indications and long-term safety of anti-VEGF therapy remain incompletely established. Concerns have been raised regarding systemic effects, technical challenges specific to neonatal administration, and the potential for late recurrence [67]. Despite these uncertainties, anti-VEGF therapy has become an established treatment option in ROP management. Reflecting this shift, clinical guidelines have been developed and updated in Japan [68].

This situation resembles the early phase of photocoagulation therapy, in which clinical adoption preceded the accumulation of definitive evidence. In Japan, the standard of care has historically been interpreted not solely on the basis of definitive evidence but through a comprehensive assessment of professional consensus, clinical practice, and availability of alternative treatments at a given time [69].

In this context, it is conceivable that, in certain clinical settings, failure to consider anti-VEGF therapy—particularly when supported by emerging evidence and clinical practice—may be regarded as a potential deviation from the standard of care. Conversely, given the persisting uncertainties regarding its long-term outcomes [70], the importance of thorough explanation and informed consent is likely to increase. Accordingly, future ROP-related litigation may increasingly focus not on whether a specific treatment was performed, but on whether the clinical decision-making for a treatment was reasonable considering contemporaneous knowledge, whether alternative treatment options were appropriately considered, and whether adequate explanation was provided to caregivers.

From a healthcare quality and risk-management perspective, historical ROP-related litigation suggests that preventable harm and medico-legal vulnerability increase when rapidly evolving practices are implemented without adequate system-level support for standardization, cross-disciplinary coordination, and transparent communication. In contemporary ROP care, risk mitigation should prioritize (i) structured screening pathways with clear responsibility and documentation, (ii) timely referral systems for severe ROP, and (iii) transparent informed-consent processes that explicitly address uncertainty and long-term outcomes, particularly in the context of anti-VEGF therapy.

These considerations highlight the continuing need to integrate evolving clinical evidence with medico-legal expectations in the management of ROP.

## 9. Discussion

This study revealed that judicial evaluations of ROP management in Japan closely paralleled the maturation of clinical practice. In the early period, when the pathophysiology of ROP was incompletely understood and effective interventions had not yet been established [5,6,55], courts generally deferred to physicians’ discretion and were reluctant to recognize negligence. In contrast, following the dissemination of systematic screening and the availability of effective treatments such as photocoagulation [57,60], judicial expectations became more stringent, and failures in screening, treatment timing, and explanation were more frequently recognized as breaches of duty. This temporal shift suggests that the standard of care in neonatal medicine is not a static construct but is dynamically shaped by the interplay between scientific evidence, clinical practice, and the availability of therapeutic options [71,72]. This perspective is consistent with broader medico-legal frameworks in which the standard of care is understood as a dynamic construct shaped by evolving clinical knowledge, professional consensus, and contextual factors [73,74]. Notably, the Japanese judicial approach did not require complete scientific certainty; rather, it relied on a contextual evaluation of what could reasonably be expected of clinicians at a given time [69]. This perspective may be particularly relevant in rapidly evolving fields such as neonatology, where clinical decisions often precede the establishment of definitive evidence [71]. Taken together, these findings suggest that the judicial evaluation of ROP reflects not only available evidence but also the stage of clinical implementation and knowledge dissemination.

The cohort-based comparison conducted in this study further supports this interpretation. The marked difference in judicial outcomes between those of infants born before and after the mid-1970s reflects the impact of the institutionalization of ROP screening and treatment [57,60]. Although the number of cases after 1975 was small, the consistency of the observed trend and the results of the post hoc power analysis provide supportive evidence that the observed shift is unlikely to be explained solely by sampling variability. Instead, it appears to represent a structural transition in how medical practice was evaluated. This analytical component should be interpreted as a structured contextual comparison rather than a formal epidemiological analysis, and its value lies in illustrating how medico-legal evaluation evolves alongside clinical practice.

These findings also have implications beyond the historical context of ROP. The introduction of anti-VEGF therapy represents a contemporary example of a treatment modality being incorporated into clinical practice before the full establishment of long-term evidence [75]. The historical experience reviewed in this study suggests that medico-legal evaluation of such innovations is likely to depend not only on the existence of evidence but also on the degree of professional consensus, extent of clinical adoption, and availability of alternative treatments. From a practical standpoint, this highlights the importance of transparent clinical reasoning, careful documentation, and thorough communication with caregivers. In particular, as evidence evolves, the process by which treatment decisions are made—including the consideration of alternatives and the explanation of uncertainties—may be as important as the choice of treatment itself.

While this analysis is based on the Japanese medico-legal context, the findings may offer broader conceptual insights into how standards of care are dynamically interpreted amid evolving medical knowledge. Future research may extend this framework to other clinical domains or healthcare systems to further examine the generalizability and applicability of these observations.

Some limitations are to be considered when interpreting these findings. First, the number of available judicial cases, particularly after 1975, was low. Second, the analysis was based on publicly available judicial decisions and may not fully capture the clinical complexity of individual cases or include cases resolved outside of court, which constitute the majority of medical dispute resolutions in Japan [76]. Third, this study was designed as a narrative and interpretative review rather than a systematic or causal analysis. Accordingly, the findings were intended to provide conceptual insights and should not be interpreted as definitive conclusions. Despite these limitations, this study offers a historically grounded perspective on how standards of care are formed and evaluated in neonatal medicine. By integrating clinical and legal viewpoints, it provides a framework for understanding not only past litigation but also potential future challenges in ROP management.

## 10. Conclusions

This study demonstrates that medico-legal evaluations of ROP management in Japan have evolved in parallel with advances in neonatal care and the dissemination of clinical knowledge. Early judicial decisions tended to defer to physician discretion, whereas later decisions increasingly recognized deficiencies in screening, treatment, and communication as breaches of duty. These findings indicate that the standard of care in neonatal medicine is dynamically shaped by contemporaneous knowledge, clinical practice, and institutional capability. This perspective is particularly relevant in rapidly evolving fields such as ROP management. With the introduction of anti-VEGF therapies, similar medico-legal challenges are likely to arise. In this context, clinicians should ensure transparent clinical reasoning, careful consideration of alternative treatment options, clear documentation of decision-making processes, and thorough communication with caregivers, including discussion of uncertainties. Overall, this study provides a framework for understanding how standards of care are developed and evaluated in neonatal medicine and highlights practical considerations to improve clinical decision-making, risk management, and patient safety.

## Figures and Tables

**Table 1:** Comparison of ROP-related malpractice cases in Japan by birth year cohort (≤1974 vs. ≥1975).

	Birth Year ≤ 1974 *n* = 111	Birth Year ≥ 1975 *n* = 13	*p* Value
Case characteristics and trial outcomes			
Gestational age, weeks (mean ± SD)	29.7 ± 1.8	28.9 ± 2.2	0.083
Birth weight, g (mean ± SD)	1340 ± 210	1230 ± 229	0.069
Bilateral blindness, % (*n*/*N*)	88.2 (96/109)	76.9 (10/13)	0.231
Judgment at first instance favorable to medical providers, % (*n*/*N*)	88.3 (98/111)	30.8 (10/13)	< 0.001
Final judicial outcome favorable to medical providers, % (*n*/*N*)	93.7 (104/111)	38.5 (5/13)	< 0.001
Judicial recognition of negligence by issue			
Excessive oxygen administration (dose and/or duration), % (*n*/*N*)	2.9 (3/103)	10.0 (1/10)	0.148
Failure or delay in ophthalmologic screening, % (*n*/*N*)	3.9 (4/102)	61.5 (8/13)	< 0.001
Failure to initiate appropriate ROP treatment, % (*n*/*N*)	5.0 (5/100)	66.7 (8/12)	< 0.001
Delay in referral or transfer, % (*n*/*N*)	7.8 (6/77)	50.0 (2/4)	0.056
Breach of duty to explain (informed consent), % (*n*/*N*)	11.3 (9/80)	33.3 (1/3)	0.002

Data are presented as mean ± SD or *n* (%). *p* values were calculated using evaluable cases only and were obtained with Student’s *t*-test or χ^2^ test, as appropriate. Percentages are expressed as *n*/*N* (%), where *N* represents the number of cases in which the relevant issue was explicitly addressed in the judgment decision. Because some court judgments contained incomplete descriptions, denominators vary across variables. A post hoc power analysis was performed based on the observed difference in the proportion of provider-favorable final judgments between infants born ≤ 1974 and those born ≥ 1975 (93.7% vs. 38.5%). With sample sizes of 111 and 13 cases, respectively, the statistical power to detect this difference (55.2%) exceeded 95%, suggesting adequate statistical power despite the limited number of post-1975 cases. Abbreviations: ROP, retinopathy of prematurity; SD, standard deviation.

## Data Availability

No new datasets were generated for this study. Data supporting the findings of this review were derived from publicly available sources, including published literature and publicly accessible judicial records cited in the reference list.

## Abbreviations

ROP	retinopathy of prematurity
VEGF	vascular endothelial growth factor
RLF	retrolental fibroplasia
